# Surgical treatment for hydrosalpinx increases the expression of integrin αvβ3 in the endometrium during the implantation window

**DOI:** 10.3892/etm.2012.610

**Published:** 2012-06-14

**Authors:** YIPING ZHONG, JIN LI, HAITAO WU, YING YING, YAFENG LIU, CANQUAN ZHOU, YANWEN XU, XIAOTING SHEN, QUAN QI

**Affiliations:** 1 Center for Reproductive Medicine, The First Affiliated Hospital, Sun Yat-Sen University, Guangzhou, Guangdong 510080;; 2 Guangdong No.2 Provincial People’s Hospital, Guangzhou, Guangdong 510000, P.R. China

**Keywords:** hydrosalpinx, integrin αvβ3, implantation window, endometrium

## Abstract

To investigate the clinical importance of increased integrin αvβ3 expression in the endometrium following the surgical treatment for hydrosalpinx, a total of 60 patients with hydrosalpinx and 30 patients with fallopian tube obstruction were recruited. In the implantation window, immunohistochemistry was performed to detect integrin αvβ3 expression in the endometrium of the hydrosalpinx patients before and after surgery and of patients with fallopian tube obstruction. In the implantation window, integrin αvβ3 expression levels in the endometrium of hydrosalpinx patients before surgery were significantly lower compared to those in patients with fallopian tube obstruction (P<0.05). However, there were no marked differences in integrin αvβ3 expression in the implantation window between hydrosalpinx patients after surgical intervention and patients with fallopian tube obstruction (P>0.05). Furthermore, for patients with hydrosalpinx, integrin αvβ3 expression levels in the implantation window were dramatically increased after surgery (P<0.05). Hydrosalpinx decreases integrin αvβ3 expression in the endometrium in the implantation window, and integrin αvβ3 may be an important factor influencing the endometrial receptivity of hydrosalpinx patients. Surgical treatment for hydrosalpinx can improve integrin αvβ3 expression in the endometrium during implantation.

## Introduction

Under the influence of steroid from the ovaries, the endometrium undergoes periodical changes. Thus, the embryos can implant in the endometrium only during the proper phase (1). Blastocyst implantation is shared by all mammals in nature and usually occurs between 3 and 6 days after fertilization, which corresponds to ∼Days 21 to 24 or ∼5 to 8 days after the peak in luteinizing hormone (LH) levels. That is to say, the embryos enter the uterus in the implantation window. The embryos and the endometrium secrete several related proteins and cytokines in a strictly spatial-temporal sequence. These proteins and cytokines recognize each other and cooperate, leading to implantation (2). These bioactive cytokines and proteins are known as markers of endometrial receptivity.

Tubal factors are the main causes of female infertility and account for ∼40% of all causes. Furthermore, the hydrosalpinx accounts for ∼10 to 30% of the tubal factors causing infertility. *In vitro* fertilization-embryo transfer (IVF-ET) was initially applied in women with tubal factor infertility. However, numerous studies have shown that the hydrosalpinx can reduce the implantation and pregnancy rates (3). The mechanisms underlying the impact of hydrosalpinx on IVF-ET are poorly understood. There is evidence that the influence of hydrosalpinx on the endometrial receptivity is one of the mechanisms (4). In the present study, the expression of integrin αvβ3 in the endometrium during the implantation window was compared between hydrosalpinx patients and those with fallopian tube obstruction, and the expression of integrin αvβ3 in the endometrium during the implantation window in hydrosalpinx patients were also compared before and after surgery. Our results may be helpful to elucidate the cause of poor outcome of hydrosalpinx patients following IVF-ET.

## Materials and methods

### Patients

A total of 60 patients with hydrosalpinx and 30 patients with fallopian tube obstruction were recruited from April 2010 to December 2010 from the Center for Reproductive Medicine of the First Affiliated Hospital of Sun Yat-Sen University (Guangdong, China).

All patients were aged <40 years and had a regular menstrual cycle. Endocrine examinations revealed normal levels and the basal body temperature was biphasic. Hormones were not administered within 6 months before the start of the study. Cases presenting with endometriosis, uterine fibroids, polycystic ovary syndrome, ovarian cancer, infertility of unknown causes, immune infertility, chronic systemic disease, sexually transmitted disease and trophoblastic disease, and cases with a positive status for smoking and drinking were excluded from the study. Cases associated with a spouse presenting with male infertility were also excluded.

### Diagnosis

Bilateral or unilateral hydrosalpinx was diagnosed by hysterosalpingography (HSG) or laparoscopy (LAP) and untrasonography. Fallopian tube obstruction was diagnosed by HSG or LAP, and ultrasonography was performed to exclude the presence of hydrosalpinx.

### Surgical intervention of hydrosalpinx

Vaginal ultrasound-guided hydrosalpinx aspiration, laparoscopic salpingostomy, laparoscopic proximal tubal ligation or laparoscopic salpingectomy was performed.

### Sample collection and processing

The LH peak was measured by using LH strip from Day 10 of the menstrual cycle. In addition, transvaginal ultrasonography and the test of serum sex hormones were also performed. At Days 7–8 after ovulation, the endometrium was collected at the bottom of the uterus by using a curette, and the samples were washed in normal saline to remove blood. Samples were fixed in fixation solution, embedded in paraffin and sectioned. Pathological examination was carried out to confirm that the endometrium was in the secretory phase. For patients with hydrosalpinx, the endometrium was collected during the implantation window before and after surgery; collection of endometrium was performed once in patients with fallopian tube obstruction.

### Immunohistochemistry

Mouse anti-human integrin αvβ3 monoclonal antibody (Abcam) (1:80) was used for immunohistochemistry which was performed according to the manufacturer’s instructions.

### Determination of findings

Five fields were randomly selected from each section at magnification ×4,000, and the integrated optical density (IOD) was determined by using the Image-Pro Plus 5.1 Chinese software.

### Statistical analysis

The IOD was expressed as means ± standard deviation (SD). Statistical analysis was performed with SPSS version 13.0. A value of two-tailed P<0.05 was considered statistically significant.

## Results

**Figure 1:**
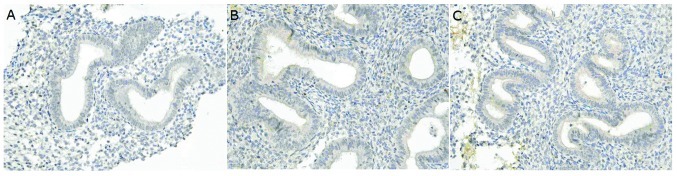
Expression of endometrial integrin αvβ3 in (A) hydrosalpinx patients before surgery, (B) hydrosalpinx patients after surgery and (C) fallopian tube obstruction patients after surgery (immunohistochemistry; magnification, ×400).

Under light microscopy, integrin αvβ3 was mainly expressed on the membrane and in the cytoplasm of the endometrial gland epithelial cells, while the endometrial interstitium had weak integrin αvβ3 expression. In hydrosalpinx patients, integrin αvβ3 expression levels were significantly different at the times before and after surgery (P<0.05). Before surgery, integrin αvβ3 expression in the endometrium of the hydrosalpinx patients (Fig. 1A) was markedly lower than that in the controls (Fig. 1C) (P<0.05). However, no dramatic difference was found in the integrin αvβ3 expression in the endometrium between hydrosalpinx patients after surgery and control patients (P>0.05) (Fig. 1B and Table I).

## Discussion

The histologically normal endometrium dos not always have a normal function and does not always reflect normal receptivity. Currently, indicators for the evaluation of the endometrium are limited. There are numerous cytokines and molecules that are being applied to evaluate the successful implantation of embryos. In the present study, we employed integrin αvβ3 as a marker of emdometrial receptivity. To date, few studies have been conducted to investigate the effect of hydrosalpinx on integrin αvβ3 expression in the endometrium.

### Biochemical characteristics of integrins

Integrins are a type of cellular adhesion molecule and are expressed on the cell membrane. They are receptors shared by the extracellular matrix and heterodimers consisting of subunits α and β in a non-covalent manner. A total of 14 α subunits and 9 β subunits have been identified and can form >20 integrins. Both subunits α and β are composed of extracellular, transmembrane and intracellular domains. The α subunit is 120–180 kDa and is indispensable for the integrin function. The β subunit is 90–110 kDa. Different combinations of α and β subunits form different integrins. The N terminal of the heterodimers of subunits α and β is extracellular and long and forms a spherical domain. In addition, the N terminal also contains a divalent cation-binding site which can specifically bind to the laminin (LN), fibronectin (FN), vitroneetin (VN) and the Arg-Gly-Asp (RGD) in the human complement C3. The C terminal is intracellular and short. Different α and β subunits have distinct structures of the C terminal. Dou *et al* (5) determined the expression levelss of α2, α3, α4, α5, α6.1, α6.2, αv, β1, β2, β3 and β5 in the endometrium during the entire menstrual cycle. They found that α2, α3 and α5 were predominantly expressed during the proliferative phase, and α4, α6.2, αv, β1, β2, β3 and β5 were mainly expressed during the secretory phase. However, α6.1 expression was constant during the entire menstrual cycle. In addition, the changes in the expression of αv and β3 in the menstrual cycle were more obvious than changes in other subunits. Moreover, different types of cells exhibit expression of different integrins. In mammals, integrins are widely expressed on the cell membrane. Currently, integrins have become an acceptable marker of endometrial receptivity.

### Role and regulation of integrins in reproduction

Integrins function via binding to the corresponding ligands. An integrin can recognize some ligands and a ligand may recognize different integrins. Integrin αvβ3 is related to endometrial receptivity and its ligands include osteopontin (OPN) (6), perlecan, FN, VN, tenascin and von Willebrand factor (vWF). During the establishment of endometrial receptivity, OPN can recognize αvβ3, which is closely related to the implantation window. During the proliferative phase, the mRNA expression of OPN is weak. During the middle or later secretory phase, the endometrial epithelial cells, lymphocytes and endometrial secretions have high mRNA expression of OPN (7). Lessey *et al* (8) proposed the ‘Sandwich model’ in the implantation of embryos, according to which the integrins expressed on the embryos and in the endometrium can bind to the OPN, which facilitates the adhesion of embryos to the endometrium. During the implantation window, the expression of integrins in the endometrium is significantly increased due to the regulation by steroids and a series of cytokines and growth factors, and the affinity of integrins is also elevated, which maximizes endometrial receptivity. At the same time, trophoblast cells in the embryos also express integrins. Thus, integrins in the endometrium and on the trophoblast cells bind to the OPN which mediates the crosstalk between embryos and endometrium. The integrins are expressed on the cell membrane in a cluster manner and the individual integrin has a low affinity to the ligands. However, the accumulated affinity of clustered integrins significantly consolidates the binding between the embryo and endometrium. Therefore, according to the ‘Sandwich model’, the endometrium finally accepts the embryo leading to endometrial receptivity. In addition, integrins may act as activators and can activate the endometrium, increase vascular permeability, promote the dilation of local blood vessels and become involved in the decidualization of endometrium, which are beneficial for the adhesion of embryos to the endometrium and subsequent implantation.

Before endometrial receptivity is established and after endometrial receptivity subsides, the expression of the estrogen receptor (ER) and progesterone receptor (PR) in the endometrial epithelial cells displays a decreasing tendency which depends on progesterone. Failure of progesterone regulation may significantly affect endometrial receptivity (9). Progesterone binds to the PR and then regulates the αvβ3 and its ligands in two ways: i) by direct regulation, where progesterone directly acts on the PR on epithelial cells of endometrium and then promotes the expression of αvβ3, OPN and other endometrial receptivity-related moleculaes (such as α1β1 and α4β1) in the epithelial cells and ii) by indirect regulation, where progesterone acts on the PR on endometrial stroma cells which stimulates the transcription of downstream genes and increases the expression of epithelium growth factor (EGF) or heparin-binding EGF-like gowth factor (HB-EGF). These factors then affect the corresponding receptors on the endometrial epithelial cells leading to the production of αvβ3 and OPN (10). At the site where the embryos implant, a series of cytokines are expressed and form a network, which can coordinate the expression of various factors which mediate endometrial receptivity. Integrins are also regulated by these factors and thus, the endometrium can achieve receptivity at the designated time. The embryos can secrete hCG and other cytokines (such as IL-1), which then bind to the corresponding receptors on the endometrium and regulate the expression of molecules in the endometrium (such as integrins) and subsequently endometrial receptivity. There is evidence showing that integrin αvβ3 binds to the RGD sequence both of which are expressed on the embryo (11). RGD may bridge the recognition between integrin αvβ3 in the endometrium and that on the embryo, but the specific mechanism is unclear. The findings above show that integrin αvβ3 is critical for the implantation of the embryo, and it is expressed not only in the endometrium but on the embryo as well.

### Effect of hydrosalpinx on integrin αvβ3 expression in the endometrium

Our results showed that integrin αvβ3 expression in hydrosalpinx patients before surgery was markedly lower than that in patients with fallopian tube obstruction. After surgical intervention for hydrosalpinx, integrin αvβ3 expression was comparable between patients in the two groups. In addition, when compared with the level in patients before surgery, integrin αvβ3 expression was dramatically increased in the hydrosalpinx patients after surgery. These findings suggest that hydrosalpinx inhibits the integrin αvβ3 expression in the endometrium during the implantation window, which is upregulated following surgical intervention. These findings were consistent with previous studies. Lessey and Castelbaum (8) found that integrin αvβ3 expression in the endometrium during the implantation window was decreased in hydrosalpinx patients, but returned to normal levels after surgical treatment for hydrosalpinx. Bildirici *et al* (12) also drew the same conclusion. Bildirici *et al* (12) investigated 10 patients with hydrosalpinx. Before surgery, the HSCORE score of integrin αvβ3 expression was <0.7 during the implantation window in 8 hydrosalpinx patients, and the mean HSCORE score was increased by 2.1 after surgery (criterion for positivity, 0.7). Statistical analysis revealed a significant difference in integrin αvβ3 expression in the endometrium of patients at a time before and after surgery. We hypothesize that hydrosalpinx may reduce endometrial receptivity via decreasing integrin expression in the endometrium.

Taken together, the endometrium undergoes periodic changes which vary among individuals. Accurate evaluation of endometrial receptivity is basic for its improvement. In the present study, we investigated the effect of hydrosalpinx on expression in the endometrium. Our results demonstrate that hydrosalpinx influences integrin αvβ3 expression in the endometrium during the implantation window, reduces the receptivity of the endometrium for the implantation of the embryo and compromises the ability to maintain pregnancy. After surgical treatment for hydrosalpinx, integrin αvβ3 expression in the endometrium is increased.

## Figures and Tables

**Table I:** Expression of endometrial integrin αvβ3 in hydrosalpinx patients and fallopian tube obstruction patients.

	Hydrosalpinx patients		Fallopian tube obstruction patients (n=30)	
	Before surgery (n=60)	After surgery (n=60)	
Integrin αvβ3	0.29±0.10	0.58±0.17	0.55±0.11
